# Genetic characterization of four dog breeds with Illumina CanineHD BeadChip

**DOI:** 10.1080/20961790.2019.1614292

**Published:** 2019-07-11

**Authors:** Zihao Yang, Jingyi Zhang, Jiashuo Zhang, Ruiyang Tao, Wei Ren, Jie Zhang, Jilin Dong, Chengtao Li, Suhua Zhang

**Affiliations:** a Department of Forensic Medicine, School of Basic Medical Science, Wenzhou Medical University, Wenzhou, China;; b Shanghai Key Laboratory of Forensic Medicine, Shanghai Forensic Service Platform, Academy of Forensic Sciences, Ministry of Justice, P.R. China, Shanghai, China;; c Department of Forensic Science, Medical School of Soochow University, Suzhou, China;; d Institute of Forensic Medicine, West China School of Basic Medical Sciences & Forensic Medicine, Sichuan University, Chengdu, China;; e Criminal Police Detachment of Qingdao Public Security Bureau, Qingdao, China

Dear Editor, Due to human's cohabitation with domesticated animals, molecular analysis of animal DNA is increasingly being admitted as evidence in forensic investigations. In 2011, recommendations from the International Society of Forensic Genetics (ISFG) for non-human DNA analysis in forensic casework were published based on the successful model for human DNA [1]. Among domesticated animals, canine DNA is perhaps the most often encountered and investigated in the forensic community [2–5]. The US, Brazil and China are the top three countries in regards to ownership of canines. Canine DNA in the form of hair, saliva, blood, urine and feces is abundant in the domestic environment and consequently is often present on evidence collected during forensic investigations. A strong need for identity identification, parentage verification and breed recognition has become apparent within the forensic community.

Short tandem repeats (STRs) analysis of canine-derived biological evidence for the identification of individuals and genetic diversity is becoming an important tool for forensic investigations [6–8]. However, for parentage testing and breed recognition, available STRs in references [6–8] and the commercial Canine ISAG STR Parentage Kit (Thermo Fisher Scientific, Waltham, MA, USA) are sometimes limited, especially for inbreeding pedigrees. In addition, some STRs (FH2613, FH2508 and FH2137) were observed with sequence block in flanking regions, resulting difficulties in genotype calling [8]. In this study, we tried to explore the canine DNA variation with single nucleotide polymorphisms (SNPs). SNPs, which are widely spread in the canine genome with lower mutation rates than STRs, are popular in canine disease association studies [9,10]. Here, we used the CanineHD BeadChip to sequence more than 170 000 SNPs. This strategy presents an average of greater than 70 markers per megabase (Mb), providing ample SNP density for analysis.

In this study, we collected EDTA-stabilized blood samples from four canine breeds, including German Shepherd (GS), Dutch Shepherd (DS), Springer Spaniel (SS) and Malinois (M). Canines were imported from European countries by the police kennel base in Qingdao, China. All individuals (*N* = 37) were authorized with studbooks. According to the studbooks, samples of the same breed were unrelated to each other. In total, we genotyped 48 samples using the CanineHD BeadChip WG-440-1001 (Illumina, Inc., San Diego, CA, USA). The sample details are attached as Supplementary Table S1. The 37 unrelated individuals investigated were GS (*n* = 12), DS (*n* = 7), SS (*n* = 7) and M (*n* = 11). Since the CanineHD BeadChip WG-440-1001 is capable of sequencing a maximum 48 samples in parallel, we also tested three DS puppies from a single brood, one negative control and seven re-sequenced samples. The seven re-sequenced samples were collected via a second blood collection, taken a year following the initial collection. The aims of the investigation were: (1) to characterize the genetic profile of the four pure dog breeds by quantifying the genetic differentiation among them and the degree of genetic homogeneity within breeds; and (2) to determine whether the results can be applied for designing breed recognition strategies aimed at distinguishing these dog breeds, as well as distinguishing the identity of individuals.

Genomic DNA (gDNA) was isolated using a QIAamp DNA Blood Kit following the manufacturer’s protocol (QIAamp; Qiagen, Hilden, German). DNA was quantified using agarose gel electrophoresis and the Nanodrop ND-200 spectrophotometer (Thermo Fisher Scientific). Detailed concentration information is listed in Supplementary Table S1. gDNA concentrations for all samples were a minimum of 50 ng/µL. DNA samples were whole-genome amplified for 20–24 h at 37 °C, fragmented, precipitated and resuspended in an appropriate hybridization buffer. The samples were hybridized on the prepared BeadChips for 16–24 h at 48 °C. Following the hybridization, nonspecifically hybridized samples were removed by washing, while the remaining specifically hybridized loci were processed for the single-base extension reaction, stained and imaged on an Illumina iScan Reader. SentrixBarcode and SentrixPosition on the chip are listed in Supplementary Table S1. We used GenomeStudio and the accompanying guidelines from Illumina (www.illumina.com) to identify individuals suitable for genetic profile analyses. Genotype data generated from the iScan system were loaded into Illumina GenomeStudio Genotyping Module and used to perform primary data analysis, including raw data normalization, clustering and genotype calling (https://support.illumina.com.cn/array/array_kits/caninehd_whole-genome_genotyping_kit/documentation.html?langsel=/cn/). A final custom report was created from GenomeStudio using PLINK Input Report, which generated a PED and MAP file to use for downstream analyses.

We evaluated the population genetic profiles using a Bayesian inference model in the program STRUCTURE 2.3.3 [11]. We used 10 000 burn-in runs, followed by 10 000 Markov Chain Monte Carlo repetitions and evaluated three possible population clusters (*K* = 2–4). Each parameter setting was repeated three times. We used STRUCTURE HARVESTER and CLUMPP v1.1.2 [12] to summarize the output, which included estimates for delta *K*, and plotted individual assignments with Distruct v1.1. The STRUCTURE approach has become a standard method of evaluating the number of genetic clusters in a dataset, while assuming equilibrium genetic conditions (Hardy–Weinberg and linkage equilibrium). These conditions may nonetheless not be fulfilled in all breeds. Therefore, we also evaluated the data with principal component analysis (PCA) methods that are without such equilibrium assumptions using the adegenet package in R 2.14.2. A phylogenetic tree was generated using Mega 7.0 (https://megasoftware.net/). The genetic differentiation between breeds was calculated using the Fst [13]. Moderate and large differentiations had Fst values ranging from 0.05 to 0.15 and 0.15 to 0.25, respectively [14].

In the PLINK Input Report, 173 662 SNPs of the 47 samples were provided, resulting in 8 162 114 genotypes, while no genotypes were called for the negative sample. The calling rate of the 47 samples ranged from 99.32% to 99.66%, while the average calling rate was 99.53%. For the seven re-sequenced samples, the both called genotypes were all consistent. However, there are some SNPs detected with genotypes in one sample, while detected with no genotype at the other double sequenced sample. This kind of sequencing error rate ranged from 0.0144% to 0.0311% and was found at 54 SNPs. These SNPs were deleted from following analysis. Among the 37 unrelated samples, data were screened with following steps: (1) max individual missing rate (mind) > 0.1; (2) removal of SNPs on the X and Y chromosomes; (3) selecting only SNPs with minor allele frequency (MAF) > 0.05; (4) removal of SNPs with pairwise genotypic associations (*r*^2^) > 0.8 within a window of 50 SNPs: PLINK command: “indep-pairwise 50 5 0.8”. The number of SNPs retained for calculations after the pruning process was 76 599.

For the 37 unrelated samples, we estimated observed heterozygosity (H_obs_) and percent polymorphic loci degree of polymorphism (P%) with the 76 599 SNPs in PLINK of each breed. The H_obs_ values differed significantly among breeds (1-way ANOVA), and all pairwise comparisons of H_obs_ were also highly significant (*P* < 0.001). The dog breeds were ranked relative to genetic variation (H_obs_ and P%) expressed as DS > M > SS > GS.

Among the 76 599 SNPs, we found some fixed SNPs with MAF equal to 0 among all the tested individuals of a breed. The number of fixed SNPs of GS, DS, M and SS is 23 729, 8 552, 15 074 and 29 634, respectively. Among these fixed SNPs, a Venn diagram (Supplementary Figure S1) was constructed with VENNY 2.1 to show SNP numbers, unique or shared, across the four breeds. Venn diagrams are illustrations composed of overlapping circles that demonstrate the relations between finite collections of breeds and are most useful in defining areas of commonality among different breeds. A breed-specific SNP was defined as “private SNP” for which one of the alleles was detected only in one breed (a fixed SNP). The number of “private SNPs” of GS, DS, M and SS is 11 494, 2 325, 5 841 and 17 329, respectively. We also validated the data with three DS puppies in a brood (sample DS-O-1, DS-O-2 and DS-O-3) (Supplementary Table S1) and found 1 882 SNPs of the 2 325 DS “private SNPs” are with fixed genotypes. These “private SNPs” which identified as specific breed markers would be helpful for breed identification or evaluation of purity of a breed. Moreover, the quantity of “private SNPs” would be minimized when more samples were further tested. Grasso et al. [14] found 99, 99, and 11 190 fixed SNPs for Corriedale, Merino and Creole sheep, respectively. Wiggans et al. [15] reported that a set of 622 SNPs can be used to determine breed identity as part of the quality control process for dairy cattle. Ramos et al. [16] reported 29 146 putative breed-specific SNPs in five pig breeds (Duroc, Landrace, Large White, Pietrain and Wild Boar). In future studies, an independent group of the aforementioned four canine breed samples should be tested for validation of the “private SNPs” reported here.

The polymorphic SNPs (MAF > 0.05) presented in GS, DS, M and SS are 52 871, 68 048, 61 525, and 46 965, respectively. Highly polymorphic SNPs (MAF > 0.4) presented in GS, DS, M and SS are 535, 1 583, 2 730 and 2 227, respectively; GS had a much lower number of highly polymorphic SNPs than the other three breeds (1%). Among these highly polymorphic SNPs, we found only 129 SNPs were observed in all the four breeds, which could be used for canine parentage testing and individual identification. We analyzed the 129 SNPs in the three DS puppies of a brood (sample DS-O-1, DS-O-2 and DS-O-3) and their parents (1-Z07-A and 1-Z08-B) and found they all follow Mendel's law. And these polymorphic SNPs can distinguish one individual from another.

With the filtered 76 599 SNPs, we performed STRUCTURE, PCA and phylogenetic tree analysis. Supplementary Figure S2(A,B) supported the presence of three genetic clusters (*K* = 3) determined by the delta *K* method. With *K* = 3, the GS, M and SS were clearly differentiated. Only DS individuals formed three clusters with high levels of admixture. Both the PCA (Supplementary Figure S2(C)) and the Neighbor-net tree (Supplementary Figure S2(D)) revealed a clear genetic separation of the four breeds. In Supplementary Figure S2(C), the four breeds were clearly differentiated by the three principle components, which sufficiently accounted for the observed population structure, with findings very similar to those provided in Supplementary Figure S2(A). Only the DS samples located within a loose cluster. In Supplementary Figure S2(D), individuals within the same breed were clustered together, and the different breeds were distributed in distant branches. The four breeds considered in this study were clearly genetically differentiated from each other, regardless of current small population sizes.

Large genetic differentiations were observed among the GS, M and SS, with Fst values ranging from 0.18 to 0.22 (Supplementary Table S2). Low-to-moderate genetic similarity has been detected when DS was compared with the three other breeds, with an Fst ranging from 0.04 to 0.14 (Supplementary Table S2). This result agrees with the above findings.

To conclude, the genetic characterization, despite the small population size, showed relatively high genetic diversity among the four dog breeds considered in this study. The results could be helpful in developing specific sets of SNPs for breed identification, individual identification and parentage testing, all of which could be used in forensics, population genetics, and other analyses.

Zihao Yang *Department of Forensic Medicine, School of Basic Medical
Science, Wenzhou Medical University, Wenzhou, China* *Shanghai Key
Laboratory of Forensic Medicine, Shanghai Forensic Service Platform, Academy of Forensic
Sciences, Ministry of Justice, P.R. China, Shanghai, China* Jingyi
Zhang *Shanghai Key Laboratory of Forensic Medicine, Shanghai Forensic
Service Platform, Academy of Forensic Sciences, Ministry of Justice, P.R. China, Shanghai,
China* *Department of Forensic Science, Medical School of Soochow
University, Suzhou, China* Jiashuo Zhang *Shanghai Key
Laboratory of Forensic Medicine, Shanghai Forensic Service Platform, Academy of Forensic
Sciences, Ministry of Justice, P.R. China, Shanghai,
China* *Department of Forensic Science, Medical School of Soochow
University, Suzhou, China* Ruiyang Tao *Shanghai Key
Laboratory of Forensic Medicine, Shanghai Forensic Service Platform, Academy of Forensic
Sciences, Ministry of Justice, P.R. China, Shanghai,
China* *Institute of Forensic Medicine, West China School of Basic
Medical Sciences & Forensic Medicine, Sichuan University, Chengdu,
China* Wei Ren *Criminal police detachment of Qingdao Public
Security Bureau, Qingdao, China* Jie Zhang *Criminal police
detachment of Qingdao Public Security Bureau, Qingdao, China* Jilin
Dong *Criminal police detachment of Qingdao Public Security Bureau, Qingdao,
China* Chengtao Li *Department of Forensic Medicine, School
of Basic Medical Science, Wenzhou Medical University, Wenzhou,
China* *Shanghai Key Laboratory of Forensic Medicine, Shanghai
Forensic Service Platform, Academy of Forensic Sciences, Ministry of Justice, P.R. China,
Shanghai, China* *Department of Forensic Science, Medical School of
Soochow University, Suzhou, China* *Institute of Forensic Medicine,
West China School of Basic Medical Sciences & Forensic Medicine, Sichuan University,
Chengdu, China* Suhua Zhang *Shanghai Key Laboratory of
Forensic Medicine, Shanghai Forensic Service Platform, Academy of Forensic Sciences,
Ministry of Justice, P.R. China, Shanghai,
China* zhangsh@ssfjd.cn
