# Supplementary material for: Genetic characterization of four dog breeds with Illumina CanineHD BeadChip
Source: Forensic Sci Res. 2019 Jul 11;4(4):354–7. doi: 10.1080/20961790.2019.1614292 (PMC7100786; doi:10.1080/20961790.2019.1614292)
Supplement: Supplemental Material [file TFSR_A_1614292_SM7095.docx]

**Supplementary Materials**

**Supplementary Figures**


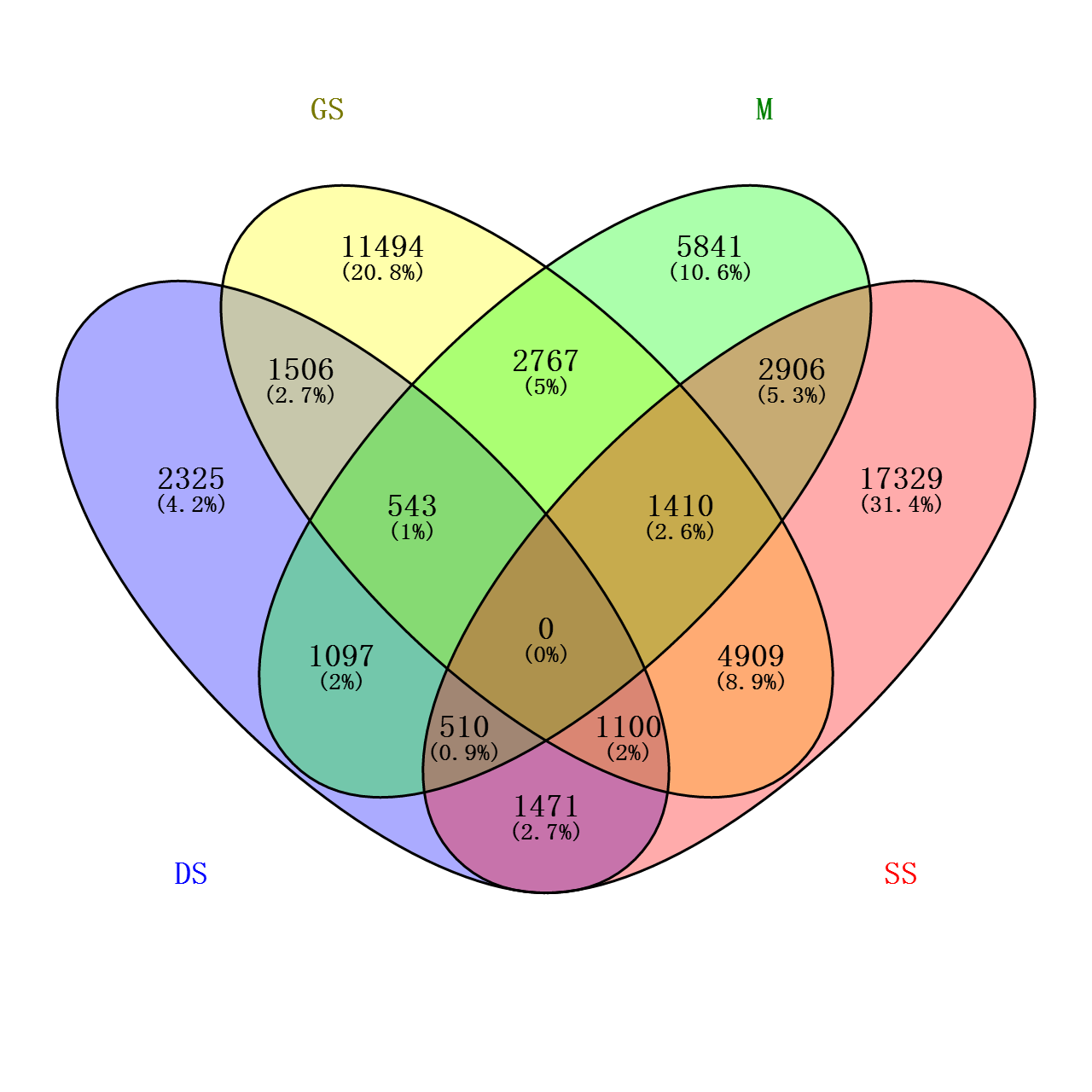


Supplementary Fig. S1 A four-way Venn diagram to show fixed SNPs (MAF = 0) in the four breeds. The numbers of SNPs shared in common are indicated at the intersections of the circles in the Venn diagram. In addition, 11494, 2325, 5841 and 17329 SNPs were recognized as "private SNPs" for German Shepherd (GS), Dutch Shepherd (DS), Malinois (M), and Springer Spaniel (SS).


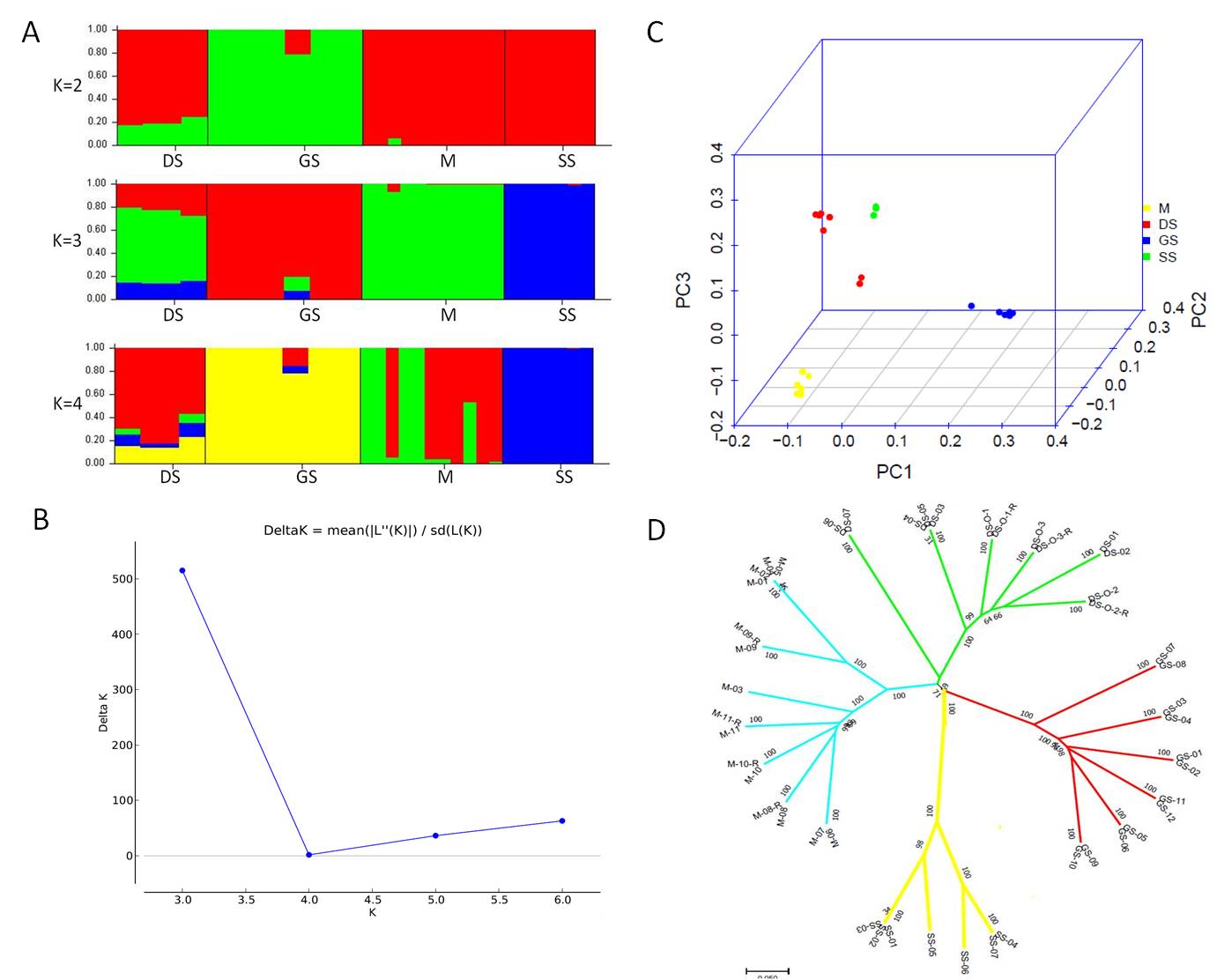


Supplementary Fig. S2 Population analysis estimated for 7,660 SNPs among unrelated individuals of the four breeds. GS=German Shepherd; DS=Dutch Shepherd; SS=Springer Spaniel; M=Malinois. (A) Proportion of membership of 37 individuals from four canine populations for K=2-4, as calculated by STRUCTURE software. (B) Multiple runs for each K were concatenated using CLUMPP, and Distruct was subsequently used to generate images. Delta K plot showing a peak at K=3. (C) Three-dimensional principle component analysis of the four breeds. Each point represents one animal, and individuals are colored according to the breed. (D) Phylogenetic tree analysis of the four breeds.

**Supplementary Tables**

Supplementary Table S1 The detail information of samples and correspondingly extracted DNAs and sequencing positions on the CanineHD BeadChip

| List | Breeds | Analysis_  ID | Sex | DNA Concentration (ng/μL) | DNA: A260/280 | DNA: A260/230 | SentrixBarcode | SentrixPosition | Note |
| --- | --- | --- | --- | --- | --- | --- | --- | --- | --- |
| 1 | German Shepherd (GS) | GS-01 | M | 64.7 | 1.80 | 1.09 | 200514100015 | R01C02 |  |
| 2 |  | GS-02 | M | 119.8 | 1.86 | 1.50 | 200514100015 | R02C02 |  |
| 3 |  | GS-03 | M | 79.0 | 1.82 | 2.04 | 200514100004 | R02C02 |  |
| 4 |  | GS-04 | M | 85.1 | 1.85 | 2.07 | 200514100012 | R02C02 |  |
| 5 |  | GS-05 | M | 284.3 | 1.90 | 1.78 | 200514100015 | R03C02 |  |
| 6 |  | GS-06 | M | 137.0 | 1.84 | 1.77 | 200514100015 | R01C01 |  |
| 7 |  | GS-07 | M | 161.5 | 1.90 | 1.83 | 200514100015 | R04C02 |  |
| 8 |  | GS-08 | M | 161.5 | 1.89 | 1.78 | 200514100015 | R05C02 |  |
| 9 |  | GS-09 | F | 317.6 | 1.92 | 1.78 | 200514100015 | R06C02 |  |
| 10 |  | GS-10 | F | 164.8 | 1.86 | 2.06 | 200514100015 | R03C01 |  |
| 11 |  | GS-11 | F | 318.3 | 1.93 | 1.91 | 200514100049 | R01C01 |  |
| 12 |  | GS-12 | F | 148.3 | 1.82 | 2.06 | 200514100015 | R04C01 |  |
| 13 | Dutch Shepherd (DS) | DS-01 | F | 213.3 | 1.90 | 1.96 | 200514100049 | R02C01 |  |
| 14 |  | DS-02 | F | 155.8 | 1.91 | 1.99 | 200514100015 | R05C01 |  |
| 15 |  | DS-03 | M | 334.4 | 1.95 | 1.85 | 200514100049 | R03C01 |  |
| 16 |  | DS-04 | M | 136.0 | 1.85 | 2.06 | 200514100004 | R04C02 |  |
| 17 |  | DS-05 | M | 248.3 | 1.85 | 2.15 | 200514100012 | R04C02 |  |
| 18 |  | DS-06 | F | 127.5 | 1.87 | 1.83 | 200514100004 | R03C02 |  |
| 19 |  | DS-07 | F | 515.1 | 1.84 | 1.91 | 200514100012 | R03C02 |  |
| 20 |  | DS-O-1 | M | 200.3 | 1.87 | 2.17 | 200514100004 | R05C01 | Offspring of sample DS-01 and DS-03 |
| 21 |  | DS-O-2 | M | 209.3 | 1.87 | 2.15 | 200514100004 | R06C01 | Offspring of sample DS-01 and DS-03 |
| 22 |  | DS-O-3 | M | 129.0 | 1.85 | 1.70 | 200514100004 | R01C02 | Offspring of sample DS-01 and DS-03 |
| 23 |  | DS-O-1-R | M | 324.2 | 1.86 | 2.09 | 200514100012 | R05C01 | Second time of blood collecting from sample DS-O-1 |
| 24 |  | DS-O-2-R | M | 301.9 | 1.87 | 2.01 | 200514100012 | R06C01 | Second time of blood collecting from sample DS-O-2 |
| 25 |  | DS-O-3-R | M | 215.5 | 1.85 | 2.11 | 200514100012 | R01C02 | Second time of blood collecting from sample DS-O-3 |
| 26 | Springer Spaniel (SS) | SS-01 | M | 290.5 | 1.90 | 1.81 | 200514100049 | R03C02 |  |
| 27 |  | SS-02 | M | 138.3 | 1.85 | 2.09 | 200514100004 | R06C02 |  |
| 28 |  | SS-03 | M | 315.3 | 1.84 | 2.10 | 200514100012 | R06C02 |  |
| 29 |  | SS-04 | F | 481.2 | 1.90 | 1.64 | 200514100049 | R04C02 |  |
| 30 |  | SS-05 | F | 125.0 | 1.82 | 2.03 | 200514100015 | R06C01 |  |
| 31 |  | SS-06 | F | 481.9 | 1.89 | 1.67 | 200514100049 | R05C02 |  |
| 32 |  | SS-07 | F | 205.8 | 1.83 | 1.91 | 200514100015 | R02C01 |  |
| 33 | Malinois (M) | M-01 | M | 459.2 | 1.91 | 1.65 | 200514100049 | R04C01 |  |
| 34 |  | M-02 | M | 383.6 | 1.91 | 1.79 | 200514100049 | R05C01 |  |
| 35 |  | M-03 | F | 401.2 | 1.88 | 1.80 | 200514100049 | R06C01 |  |
| 36 |  | M-04 | F | 118.0 | 1.85 | 2.07 | 200514100004 | R05C02 |  |
| 37 |  | M-05 | F | 376.1 | 1.83 | 2.01 | 200514100012 | R05C02 |  |
| 38 |  | M-06 | F | 330.5 | 1.90 | 1.81 | 200514100049 | R01C02 |  |
| 39 |  | M-07 | F | 313.4 | 1.90 | 1.63 | 200514100049 | R02C02 |  |
| 40 |  | M-08 | F | 132.8 | 1.81 | 1.54 | 200514100004 | R01C01 |  |
| 41 |  | M-09 | M | 383.3 | 1.86 | 2.07 | 200514100004 | R02C01 |  |
| 42 |  | M-10 | M | 93.5 | 1.87 | 1.89 | 200514100004 | R03C01 |  |
| 43 |  | M-11 | F | 177.5 | 1.85 | 2.04 | 200514100004 | R04C01 |  |
| 44 |  | M-08-R | F | 109.5 | 1.84 | 2.19 | 200514100012 | R01C01 | Second time of blood collecting from sample M-08 |
| 45 |  | M-09-R | M | 274.7 | 1.85 | 2.20 | 200514100012 | R02C01 | Second time of blood collecting from sample M-09 |
| 46 |  | M-10-R | M | 165.9 | 1.85 | 2.05 | 200514100012 | R03C01 | Second time of blood collecting from sample M-10 |
| 47 |  | M-11-R | F | 144.2 | 1.84 | 2.02 | 200514100012 | R04C01 | Second time of blood collecting from sample M-11 |
| 48 |  | Neg | / | / | / | / | / | / | Negative control |
| Sex column: F=Female; M=Male | |  |  |  |  |  |  |  |  |

| Supplementary Table S2 Genetic differentiation between breeds, expressed as the fixation index (Fst) | | | | | |
| --- | --- | --- | --- | --- | --- |
| Canine breeds | Fst | | | | |
|  | German shepherd | Dutch Shepherd | Malinois | Springer spaniel |  |
| German shepherd | / |  |  |  |  |
| Dutch Shepherd | 0.14 | / |  |  |  |
| Malinois | 0.19 | 0.04 | / |  |  |
| Springer spaniel | 0.18 | 0.13 | 0.22 | / |  |

**Word count= 3247**
